# Survival and prognostic factors for adrenocortical carcinoma: a single institution experience

**DOI:** 10.1186/s12894-015-0038-1

**Published:** 2015-05-27

**Authors:** Zlatibor Loncar, Vladimir Djukic, Vladan Zivaljevic, Tatjana Pekmezovic, Aleksandar Diklic, Svetislav Tatic, Dusko Dundjerovic, Branislav Olujic, Nikola Slijepcevic, Ivan Paunovic

**Affiliations:** Emergency Centre, Clinical Centre of Serbia, Faculty of Medicine, University of Belgrade, Pasterova 2, 11000 Belgrade, Serbia; Centre for Endocrine Surgery, Clinical Centre of Serbia, Faculty of Medicine, University of Belgrade, Pasterova 2, 11000 Belgrade, Serbia; Institute of Epidemiology, Faculty of Medicine, University of Belgrade, Visegradska 26A, Belgrade, 11000 Serbia; Institute of Pathology, Faculty of Medicine, University of Belgrade, Dr Subotica 1, 11000 Belgrade, Serbia

**Keywords:** Adrenal gland, Cortex, Carcinoma, Surgery, Survival, Mitotane

## Abstract

**Background:**

Adrenocortical carcinoma (ACC) is aggressive, but rare tumours that have not been sufficiently studied. The aim of our study was to present the demographic and clinical characteristics of patients with ACC, to determine the overall survival rates, analyse the effect of prognostic factors on survival, as well as to identify favorable and unfavourable predictors of survival.

**Method:**

The study included 72 patients (42 women and 30 men) with ACC. We analysed the prognostic value of the demographic and clinical characteristics of the patients, tumour characteristics, therapy administered and survival rates. Kaplan-Meier survival curves and the log-rank test were used to estimate the overall and specific survival probabilities and the Cox regression model was used to identify independent prognostic factors for survival.

**Results:**

The patients had mean age of 50 years. The 1-, 5-, and 10-year probabilities of survival in patients with ACC were 52.5 %, 41.1 %, and 16.4 %, respectively. The median survival time was 36 months. The results of multivariate Cox regression analysis showed that the presence of lymphatic metastases (HR = 7.37, 95 % CI = 2.31-23.48, p = 0.001) and therapy with mitotane (HR = 0.11, 95 % CI = 0.04-0.27, p = 0.001) were independent prognostic factors for survival.

**Conclusion:**

The presence of lymphatic metastasis is an unfavourable prognostic factor, while postoperative therapy with mitotane is a favorable prognostic factor for survival in patients with ACC.

## Background

The more frequent clinical use of ultrasound and computerized tomography have increased the detection of adrenal tumours. Such adrenal incidentalomas are usually benign adenomas. Adrenocortical carcinoma (ACC) is rare, but aggressive malignant endocrine tumour. An ACC is the second most aggressive endocrine tumour, after anaplastic thyroid cancer. In addition, an ACC is the second rarest cancer of the endocrine system, following parathyroid cancer. The annual incidence of ACC is one per million population and ACC is responsible for 0.2 % of all cancer-related mortality [1, 2]. Even though a multimodal approach is used in the treatment of these patients, in which surgery has the most important role, the prognosis for these patients is poor. Most ACC occur as sporadic tumours, but ACC can be part of the rare hereditary Li-Fraumeni syndrome. The pathogenesis of ACC is not well-known. In agreement with the characteristics of benign tumours, ACC can be hormonally-active (functional) or hormonally-inactive (non-functional). Approximately 70 % of all ACC are hormonally active, and present in greater than one-half of cases with Cushing's syndrome [3, 4]. The most common first sign of a hormonally-inactive ACC is abdominal pain [4]. Because ACC is very rare tumour there are not many institutions that have had much experience with these tumours, and therefore clinical characteristics, optimal treatment approaches, prognosis, and prognostic factors are still in need of research.

The aims of our study were as follows: present the demographic and clinical characteristics of patients with ACC; determine the overall survival rates; analyse the role of prognostic factors on survival, and identify favourable and unfavourable predictors of survival.

## Methods

This cohort study included 72 consecutive patients who were diagnosed with ACC based on definitive histopathologic findings and who underwent operative treatment at the Clinical Centre of Serbia between 1996 to 2014. The following data were collected from patient records, a specialized database, and other medical documentation: basic demographic characteristics of the patients (gender and age); clinical characteristics (stage of the disease, size and weight of the tumour, presence of lymphatic and distant metastases, tumour localization [i.e., left vs. right side and local tumour infiltration of surrounding tissues]; operative treatment (type of operation, surgeon who performed the surgery [i.e., as a prognostic factor based on experience], re-operation for local recurrence of the disease, and operative treatment of distant metastases); other modes of therapy (radiotherapy and chemotherapy); hormonal activity of the tumour (functional, or non-functional); and type of hypersecretion of functional tumours.

ACC was classified into one of four stages. The first stage included patients with a tumour sized ≤ 5 cm in diameter and without local or distant metastases. The second stage included patients with a tumour ≥ 5 cm in diameter and without infiltration of surrounding tissues, or local or distant metastases. The third stage included patients with a tumour of any size but with infiltration of surrounding tissue or local lymphatic metastasis. The fourth stage included patients with tumours of any size, but with infiltration of surrounding tissues and local lymphatic metastases or distant metastases only. Continuous variables (age, tumour weight, and tumour size) were transformed into dichotomous variables based on distribution. Other variables (stage of disease, surgical approach, and type of operation) were transformed into dichotomous variables. Surgical approaches were categorized as extraperitoneal or transperitoneal.

After analysing the stages of disease individually, we grouped stages I and II together and stages III and IV together. For the type of operation, we formed two groups also. The first group included patients with incomplete resection of the tumour (i.e., a sub-adrenalectomy). The second group included patients who had an adrenalectomy with or without resection of surrounding tissues.

Mitotane was the only form of chemotherapy, and when used, the dosage was 4 g/d. Hormonal activity was measured for all patients pre-operatively. All surgical procedures were performed by surgeons in highly specialized tertiary referral centres. Information on whether or not the patient was alive, and if not, the date of death, were retrieved through contact with the patients themselves, members of their families, and patient’s general practitioner. Only patients with a cancer-specific cause of death were included in the probability of survival calculation. Median follow-up was 48 months.

The study was approved by the Ethical Committee of the Faculty of Medicine of the University of Belgrade and carried out in compliance with the Helsinki declaration. Participants signed an informed consent prior to enrolment in the study.

### Statistical analysis

Kaplan-Meier survival curves and the log-rank test were used for determining overall survival and the specific probability of survival for each of the observed variables, respectively. We then performed univariate Cox regression analysis to determine which variables were significantly associated with length of survival. The variables which were significantly associated with length of survival at a p < 0.05 level of significance, were included in the multivariate regression analysis model to determine independent prognostic factors of survival.

## Results

As shown in Table 1, ACC occurred in women nearly 50 % more often than men.

**Table 1:** Basic demographics and clinical characteristics of patients with ACC

Variable	Number	Percent
Gender		
Females	42	58.3
Males	30	41.7
Age		
<20	2	2.8
21–30	4	5.6
31–40	7	9.7
41–50	24	33.3
51–60	19	26.4
61–70	16	22.2
70+		
Stage of disease		
I	2	3.1
II	31	48.4
III	20	31.3
IV	11	17.2
Tumour size		
<100	43	71.7
101+	17	28.3
Tumour weight		
<300	39	69.6
301+	17	30.4
Lymphatic metastasis		
Yes	8	12.1
No	58	87.9
Distant metastases		
Yes	6	8.8
No	62	91.2
Local infiltration		
Yes	29	42.6
No	39	57.4
Surgical approach		
Subcostal laparotomy	48	66.7
Transdorsal lumbotomy sec.Young	16	22.2
Median laparotomy	1	1.4
Bilateral subcostal laparotomy	3	4.2
Lumbotomy sec. Kifer	3	4.2
Transrectal laparotomy	1	1.4
Tumour localization		
Left side	34	47.2
Right side	37	51.4
Bilateral	1	1.4
Type of operation		
Biopsy	5	7.1
Tumour reduction	3	4.3
Adrenalectomy	50	71.4
Extended adrenalectomy	12	17.1
Surgeons experience		
Specialist up to 10 years	21	29.2
Specialist over 10 years	51	70.8
Mitotane		
Yes	30	63.8
No	17	36.2
Hormonal activity		
Functional	19	26.4
Afunctional	53	73.6
Clinical presentation		
Asymptomatic	28	38.9
Symptomatic	44	61.1

The highest number of ACC occurred in patients in their sixth decade of life; the youngest patient with an ACC was 17 years of age, while the oldest patient was 72 years of age, and the mean age was 50.4 years. Nearly one-half of the patients had stage II disease; patients with stage I disease were extremely rare. Approximately 70 % of patients had a tumour ≤ 10 cm in diameter weighing ≤ 300 gram. At time of diagnosis, ACC lymphatic metastases were present in 12 % of patients, while distant metastases were present in six patients (lung, n = 3; liver, n = 2; contralateral adrenal gland, n = 1. Local tumour invasion was present in ≥ 40 % of patients, which demonstrates the aggressive nature of the tumours. One patient presented with an IVC thrombus. Two-thirds of patients were operated through a subcostal laparotomy approach and 22 % through a transdorsal approach; other approaches were rarely used. An endoscopic approach was not used for this group of patients. There was a similar rate of tumour occurrence on the right and left sides. Most patients (88 %) underwent potentially radical surgery, while tumour reduction or biopsy was performed in one of nine patients. All operations were performed by one of seven specialist surgeons, and ≥ 70 % of the operations were performed by a surgeon with 10 years of experience. Transcutaneous radiotherapy was administered to only one patient, while two-thirds of the patients received chemotherapy with mitotane. Following primary surgery, over a period of 1–6 years, 6 patients underwent re-operation for local tumour recurrence. In addition, two patients underwent surgery for distant metastases to the lungs during this time period. Most of the tumours (73.6 %) were not hormonally-active. Of the 19 patients with functional tumours, 16 had Cushing’s syndrome (hypersecretion of cortisol) one had hypersecretion of sex hormones, and two had mixed hypersecretion of cortisol and sex hormones. Most patients had clear symptoms at diagnosis, while nearly 40 % of patients were asymptomatic with general symptoms such as weight loss, anaemia or fatigue. Of the 44 symptomatic patients, pain was the predominant symptom in 25, while 19 had clinical manifestations of hypersecretion of cortisol (hypertension, hirsutism, amenorrhea).

**Fig. 1:**
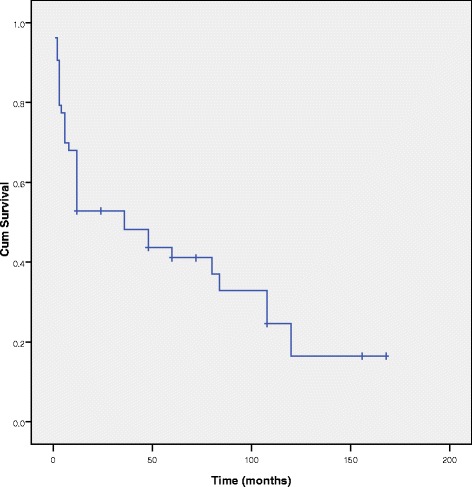
Kaplan-Meier survival curve for ACC

Figure 1 shows the overall probability of survival for patients with ACC. The 6-month, 1-year, 3-year, 5-year, 10-year probabilities of survival were 69.8 %, 52.5 %, 48.2 %, 41.1 %, and 16.4 %, respectively. The median survival time was 36 months (95 % CI = 13.4–58.5) and the mean survival time was 61.5 months (95 % CI = 42.7–80.1).

The results of univariate Cox regression analysis are presented in Table 2. The variables significantly associated with patient survival include gender, age, disease stage, tumour weight, lymphatic and distant metastases, local tumour invasion, surgical approach, and therapy with mitotane.

**Table 2:** Results of univariate Cox regression analyses

Variable	P	HR	95 % CI
Gender (male vs. female)	0.028	2.17	1.09–4.32
Age (years) (50+ vs. <50)	0.015	2.40	1.18–4.86
Stage of disease (III–IV vs. I-II)	0.005	2.94	1.39–6.21
Tumour size (mm) (100+ vs. <100)	0.437	1.36	0.62–2.99
Tumour weight (gram) (300+ vs. <300)	0.047	2.26	1.01–5.05
Lymphatic metastasis (present vs. absent)	0.001	4.86	1.88–12.54
Distant metastases (present vs. absent)	0.001	4.31	1.45–12.83
Local infiltration (present vs. absent)	0.001	3.20	1.57–6.52
Surgical approach (extraperitoneal vs. transperitoneal)	0.022	0.29	0.10–0.84
Localization (right vs. left)	0.251	1.50	0.75–3.01
Type of surgery (biopsy and tumor reduction vs. adrenalectomy and extended adrenalectomy)	0.191	0.52	0.20–1.38
Reoperation (yes vs. no)	0.550	0.75	0.29–1.95
Surgeons experience (10+ vs. <10)	0.704	0.86	0.40–1.85
Mitotane (used vs. not used)	0.001	0.13	0.06–0.31
Hormonal activity (non-functional vs. functional)	0.544	1.28	0.57–2.86
Symptomatic presentation (yes vs. no)	0.536	0.81	0.41–1.60

HR–Hazard ratio; 95 % CI - Confidence interval

The results of multivariate Cox regression analysis, which included all variables that were associated with patient survival at a p < 0.05 level of significance, are shown in Table 3. The independent prognostic factors of patient survival include lymphatic metastases (hazard ratio [HR] = 7.37, 95 % CI = 2.31–23.48) as an unfavourable prognostic factor, and therapy with mitotane (HR = 0.11, 95 % CI = 0.04–0.27), as a favourable prognostic factor.

**Table 3:** The prognostic factors for survival in patients with ACC (multivariate Cox regression analysis)

Variable	p	HR	95 % CI
Gender (male vs. female)	0.918	1.05	0.43–2.58
Age (years) (50+ vs. <50)	0.593	1.31	0.49–3.54
Stage of disease (III–IV vs. I-II)	0.879	1.15	0.20–6.71
Tumour weight (g) (300+ vs. <300)	0.665	1.35	0.34–5.36
Lymphatic metastasis (present vs. absent)	0.001	7.37	2.31–23.48
Distant metastases (present vs. absent)	0.491	1.82	0.33–10.09
Local infiltration (present vs. absent)	0.306	1.64	0.64–4.23
Surgical approach (extraperitoneal vs. transperitoneal)	0.381	0.55	0.15–2.09
Mitotane (used vs. not used)	0.001	0.11	0.05–0.27

HR–Hazard ratio; 95 % CI - Confidence interval

## Discussion

Data on the incidence of ACC are limited, but there has been no increase in incidence, even though there has been an increase in the number of diagnosed adrenal tumours and the number of adrenal operations. This increase in the early diagnosis and operative treatment of adrenal tumours could in fact be the reason for the slight decrease in the incidence of ACC in Holland over the past 20 years; the incidence has declined from 1.3 to 1.0 per million inhabitants [2].

The gender prevalence in our study corresponds to data from the literature. Women are affected by ACC more often than men, with a ratio of 1.2–1.5:1 [1–4]. Even though most patients are in their fifth or sixth decade of life at the time of diagnosis, with a mean age of 43–56 years, ACC occurs in all age groups, including children [1–4]. The population-based age-standardized incidence rate for patients <20 years of age is 0.2 per million person-years [5].

ACC is rarely diagnosed as stage I. Indeed, in the current study study only 3 % of patients were diagnosed in stage I, compered to 6 % according to the literature [3, 6, 7]. Most patients (approximately 50 %) are diagnosed with stage II ACC, as was the case in the current study and published data [3, 4, 7]. ACC tumours are usually large in size. The mean size of ACC tumours according to published data is ≥ 10 cm [1, 6]. In the current study, the size of ACC tumours ranged from 3.5 to 23 cm (mean, 9.8 cm; standard deviation [SD], 4.0 cm), and the weight ranged from 15 to 2450 g (mean, 323 g; SD, 481 g). Lymphatic metastases are present in 20 % of patients with ACC at the time of diagnosis, while distant metastases occur in nearly 30 % of patients with ACC [1].

The most common sites for distant metastases are in lungs and liver [8]. Even though, lymphatic metastases are often present, locoregional lymph node dissection is not routinely performed, although Reibetanz et al. suggested that locoregional lymph node dessection improves oncologic outcome [9].

The optimal treatment plan for ACC has not been well-defined. The best results have been achieved with surgical treatment, which has the most important role in the treatment of ACC, while additional treatment options are still a matter of discussion [10]. Surgical treatment is relatively safe, considering that the peri-operative mortality is approximately 5 % [3]. A subcostal laparotomy is the most common approach to ACC; a laparoscopic approach was not used at our institution, even though we perform laparoscopic surgery for other indications. Ferreira et al. also reported that a subcostal extended incision is the best approach for ACC and that it can be used even for ACC tumours ≥ 15 cm in size [11]. Laparoscopic adrenalectomy for ACC is associated with higher recurrence rates, particularly peritoneal recurrences. For this reason, open adrenalectomy is a better choice because of the oncologic benefit that surpasses the short-term benefits of minimally invasive surgery [12]. Miller et al. reported the mean size of laparoscopically-removed tumours to be 7 cm, whereas the size of tumours removed through open adrenalectomy was 12 cm. In the same study, positive margins of resection were present in 50 % of laparoscopic operations and ≤ 20 % in open adrenalectomies. Furthermore, there was a shorter interval before recurrence after laparoscopic surgery compared to open surgery (9 months vs. 19 months). For all of these reasons, Miller et al. concluded that laparoscopic surgery should not be attempted for ACC [13].

Open adrenalectomy is superior to laparoscopic adrenalectomy because of a more complete resection of the tumour [14]. Brix et al. after analyzing 35 laparoscopic and 117 open adrenalectomies, concluded that for localized ACC tumours ≤ 10 cm in diameter, laparoscopic adrenalectomy performed by an experienced surgeon is not inferior to open adrenalectomy [15].

ACC is considered a radioresistant tumour, thus radiotherapy is rarely used; except as adjuvant radiotherapy to the tumour bed in patients with incomplete tumour resection or ACC metastases as a palliative measure [16]. Data on the results of the application of adjuvant radiotherapy to the tumour bed in patients with complete resection of the tumour are limited; and the results of the effect of such adjuvant radiotherapy on reducing high rates of local recurrence of ACC are controversial [17, 18].

In the current study, the 1-, 3-, 5-, and 10-year survivals in patients with ACC were 52.5 %, 48.2 %, 41.1 %, and 16.4 %, respectively; the median survival was 36 months and the mean survival was 61.5 months. Bilimoria et al. reported a median survival of 32 months and a 5-year survival of 38 % in patients with ACC, with no evident change in survival rates between 1985 and 2000 [1]. Tritos et al. reported a median survival of 17 months in patients with ACC [19]. Tauchmanova et al. found an overall survival of 41 months in patients with ACC [4]. Schulick et al. showed a median survival of 38 months and a 5-year survival of 37 % for patients with ACC [20]. According to Keskin et al. the median survival for patients with ACC was 18 months, while the 1-year survival was 73 % and the 5-year survival was 48 % [6]. In the current study, the presence of lymphatic metastasis was a negative prognostic factor, while post-operative therapy with mitotane was a positive prognostic factor of survival for patients with ACC. Keskin et al. reported that the absence of lymphatic metastasis was a favourable prognostic factor for patients with ACC [6]. Also, Keskin et al. showed that the absence of distant metastases and an early stage of the disease were favourable prognostic factors. Additionally, Keskin et al. found gender to be a favourable prognostic factor, because survival length was five times longer in men than women (58 months vs. 12 months). Based on a multivariate analysis, Bilimoria et al. demonstrated a high risk of death with an increase in age, involved margins, and nodal or distant metastasis [1]. The presence of lymphatic metastases in patients with ACC is stage III disease. The higher the stage of disease, the worse the survival rates. Kerkhofs et al. reported, a mean survival in patients with ACC of 159 months for stage I and II disease, 26 months for stage III disease, and 5 months for stage IV disease [2]. Gomez Rivera et al. reported a mean survival in patients with ACC of 67 months for stage II disease, 13 months for stage III disease, and 3 months for stage IV disease [21]. Furthermore, Gomez Rivera at al. concluded that prognostic factors that worsen survival are older age, distant metastases, non-surgical treatment and a locally invasive tumour that involves large veins. Even in stage IV disease, better survival is expected if an ACC is resected in toto (R0), but the question that arises is whether or not there are really negative resection margins in stage IV disease [8, 22]. Dong et al. do not recommend surgical treatment for stage IV ACCbecause the prognosis is not affected; in contrast, surgical treatment in stage I and II ACC are most effective, but surgery is also recommended for stage III ACC [7].

The worst prognosis is expected when the tumour invades large veins (inferior vena cava and renal veins), which shorten disease-free interval and survival six-fold compared to patients in whom invasion of veins is not present [23]. Peri-operative mortality is 13 % when the inferior vena cava is infiltrated, but experienced surgeons should aim for a radical operation even in these cases [24]. We did not show that the extent of surgery influenced the survival outcome, even though this is considered the most important factor with respect to survival of patients with ACC. Because most of our patients had complete resection of the tumour, it was not possible to statistically prove that surgery influenced survival outcome. Based on the results of regression analysis, Tritos et al. showed that the absence of metastases at the time of diagnosis, patients ≤ 54 years of age, and complete surgical resection are independent prognostic factors for improved survival in patients with ACC [19]. Resection for cure is reported in 50–75 % of patients with ACC [3, 7]. The outcome of ACC patients is influenced by the expertise of the surgeons and number of patients at the institution where the patients undergo surgery. For this reason, Hermsen et al. emphasize the relevance of national cooperation and centralized surgery for ACC [25]. Furthermore, Lombardi et al. classified institutions into high and low-volume centres [26]. High-volume centres annually perform more than ten adrenalectomies for ACC, and the outcomes are better in such centres. The survival benefit is not only the consequence of expert surgical treatment, but also the result of a multidisciplinary approach to ACC which is practiced in these specialized centres [27]. We could not statistically analyse this parameter, because all patients underwent surgery at the same centre, which is in fact the centre where most patients with ACC are surgically treated in our country.

The use of mitotane is still under debate, although mitotane is used in greater than one-half of patients with ACC [3]. Mitotane is usually used as monotherapy, at a high-dose, which is favourable [28]. Terzolo et al. reported that the application of mitotane extends the recurrence-free interval in radically-resected ACC patients [29]. Icard et al. concluded that mitotane is only beneficial for ACC patients who undergo complete resection of the tumour [3]. In contrast, Grubbs et al. reported that the recurrence-free interval is nearly the same for patients with ACC who underwent surgery and received mitotane and patients who did not receive mitotane [30]. The hormonal activity of the tumour also influences the outcome of patients with ACC. According to Berruti et al. hypercortisolism is a prognostic factor in completely resected ACC with respect to overall survival and recurrence-free survival [31]. Icard et al. reported that precursor-secreting tumours influenced outcome [3].

ACC has a high recurrence rate. Analysing 101 re-operations for ACC, Erdogan et al. reported prolonged survival for R0 resection, even if > 1 year elapsed between the primary operation and recurrence of the disease [32].

The reported responses to conventional treatment of ACC have not been favourable. Additionally, an alternative approach, such as a wide array of chromosomal, genetic, molecular, and immunohistochemical markers, has been tested in ACC to identify reliable diagnostic and prognostic factors [33–35]. Therefore, certain molecular markers, such as the IGF system, the Wnt pathway, and p53, may be considered as potential targets for treatment and available therapeutic options [33].

There were several limitations to the present study. It would be useful if this study could be conducted as a multicentric study with a higher number of patients, because it would allow better analysis of variables with low occurrences. Additionally, there were missing data for some variables. In our study histopathological and immunochemical parameters were not included, and which will be presented in forthcoming publication.

## Conclusion

In conclusion, the presence of lymphatic metastasis at the time of diagnosis was a negative prognostic factor for survival, while postoperative therapy with mitotane was a favourable prognostic factor for survival in patients with ACC.

## Abbreviations

ACC: Adrenocortical carcinomas
CI: Confidence interval
HR: Hazard ratio
SD: Standard deviation
